# Differential Gene and MicroRNA Expression between Etoposide Resistant and Etoposide Sensitive MCF7 Breast Cancer Cell Lines

**DOI:** 10.1371/journal.pone.0045268

**Published:** 2012-09-18

**Authors:** Karobi Moitra, Kate Im, Katy Limpert, Alexander Borsa, Julie Sawitzke, Rob Robey, Naoya Yuhki, Ram Savan, Da Wei Huang, Richard A. Lempicki, Susan Bates, Michael Dean

**Affiliations:** 1 Laboratory of Experimental Immunology, Cancer and Inflammation Program, Frederick National Laboratory for Cancer Research, Frederick, Maryland, United States of America; 2 Medical Oncology Branch, Molecular Therapeutics Section, National Cancer Institute, Bethesda, Maryland, United States of America; 3 Department of Epidemiology, Bloomberg School of Public Health, Johns Hopkins University, Baltimore, Maryland, United States of America; 4 Laboratory of Immunopathogenesis and Bioinformatics, Clinical Services Program, SAIC-Frederick, Frederick, Maryland, United States of America; University of Cambridge, United Kingdom

## Abstract

In order to develop targeted strategies for combating drug resistance it is essential to understand it’s basic molecular mechanisms. In an exploratory study we have found several possible indicators of etoposide resistance operating in MCF7VP cells, including up-regulation of ABC transporter genes, modulation of miRNA, and alteration in copy numbers of genes.

## Introduction

Major advances have been made in the treatment of breast cancer; however, it still remains the leading cause of cancer death among women worldwide [1]. In the United States breast cancer is the second leading cause of cancer death among women after lung cancer [2]. The chemotherapeutic drug etoposide is used as a salvage treatment for advanced stage breast cancer and causes DNA damage by stabilization of DNA topoisomerase II. Etoposide stabilizes the topoisomerase II –DNA covalent complex impairing the strand-rejoining activity of the enzyme causing double stranded DNA breaks to persist instead of being repaired [3]. Etoposide can delay progression of the cell cycle through the late S or early G2 phase but has no effect on tubulin assembly [3]. As a single agent, oral etoposide response rate for breast cancer was found to be around 35% [4] and the mean bioavailability of orally administered etoposide is approximately 50% [3]. In a more recent study, it was found that oral etoposide in combination with cisplatin was much more effective in patients with advanced breast cancer (pre-treated with anthracyclines) than paclitaxel [5]. Etoposide (in combination with other drugs) is also widely used for the treatment of small cell lung cancer, lymphoma, leukemia and testicular cancer among others [6]. Though etoposide is widely used as therapy for cancer patients, the fact remains that tumors often acquire resistance to the drug. The nature of drug resistance is inherently multi-factorial involving mechanisms which include alteration in drug targets, inactivation/detoxification of the drug, decreased drug up take, increased drug efflux and the disregulation of the apoptotic pathway [7]. Apart from these mechanisms, in recent years, it has been found that small, non-coding RNAs called microRNAs (miRNAs) may also have a role in regulating drug resistance. miRNAs are small (18–22 nucleotide) non-coding RNAs that are capable of silencing the expression of certain genes by binding to complementary sites in the 3′UTR of these genes and causing either mRNA cleavage or translational repression [8]. miRNA abnormalities have become an emergent theme in cancer research. It was found that global miRNA expression patterns could classify human cancers according to developmental lineage and differentiation status much more accurately than mRNA expression profiling [9]. Gerard Wright first proposed the term ‘resistome’ to denote the genes responsible for antibiotic resistance and their precursors in bacteria [10], [11]. Similar to the ‘integrated network of antibiotic resistance elements’ in bacteria that provide protection against chemical threats [11], some cancer cells may also possess an integrated network that protects them from chemotherapeutic agents. In our study we identified several indicators that may represent the resistance network specific to an etoposide resistant MCF7VP breast cancer cell line. This network may contain genes and miRNA related to multiple mechanistic pathways of drug resistance, however, the applicability of the study to a clinical setting remains to be determined.

## Results

### The MCF7VP Cell Line Shows a Higher Level of Resistance to Etoposide Compared to the Drug Sensitive Parental MCF7 Cell Line

**Figure 1:**
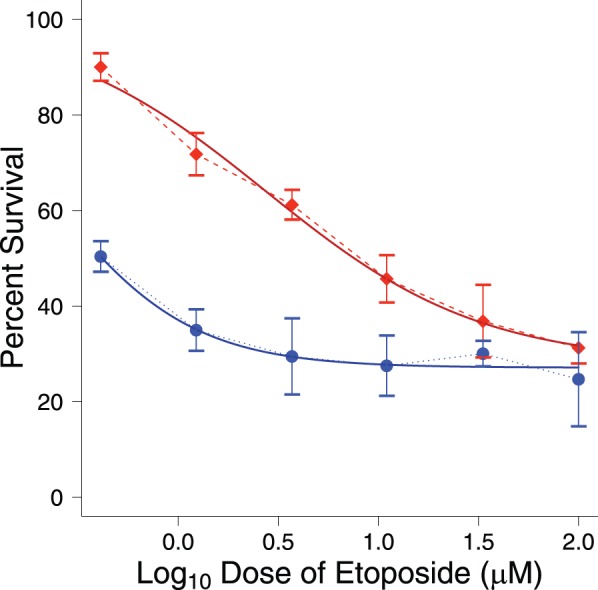
Cellular cytotoxicity assays determined that MCF7VP cells are more resistant to etoposide than the parental MCF7 cells. Curve fitting of cellular cytotoxicity data with GraphPad prism. (MCF7VP- red squares and MCF7- blue circles). The dotted line represents the actual curve and the solid line depicts the fitted curve. The assay was carried out using 10,000 cells per well of a 96 well plate and 100 µl of various concentrations (0–100 µM) of etoposide (the graph depicts log concentrations of etoposide).

To determine the extent of etoposide resistance in both the drug sensitive and drug resistant cell lines we measured sensitivity using a cellular cytotoxicity assay. Briefly, 10,000 cells per well of a 96 well plate were plated and pre-incubated at 37 degrees C for 24 hrs after which 100 µl of various concentrations (0–100 µM) of etoposide was added. After 72 hr incubation 10 µl of CCK8 reagent was added. Two hours later the absorbance was measured at 450 nm. This data was fitted to a dose-response curve using GraphPad Prism and EC50’s were calculated revealing that the MCF7VP cells were 12.5 fold more resistant to etoposide than the drug sensitive MCF7 cells (Fig. 1).

### *ABCC1* and *ABCC6* Genes are Overexpressed in the MCF7VP Cell Line

**Figure 2:**
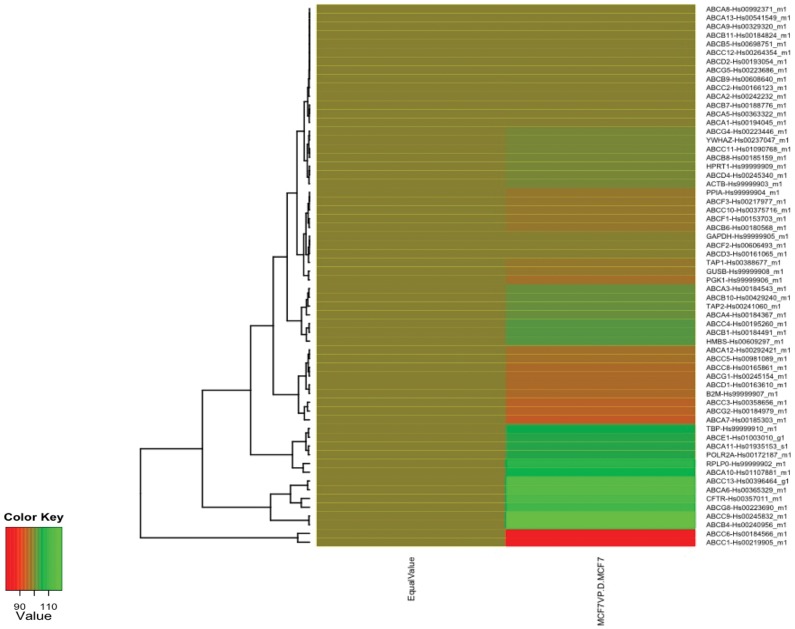
Heat map depicting the upregulation of *ABCC6* and *ABCC1* genes in MCF7VP cells. It can be seen that both *ABCC6* (red) and *ABCC1* (red) genes are upregulated in the MCF7VP cell lines. cDNA from the MCF7 and MCF7VP cells was used to run the Taqman Low Density ABC transporter arrays. The data was analyzed using Microsoft excel and R-Bioconductor to generate heat maps of the ABC array data.

**Figure 3:**
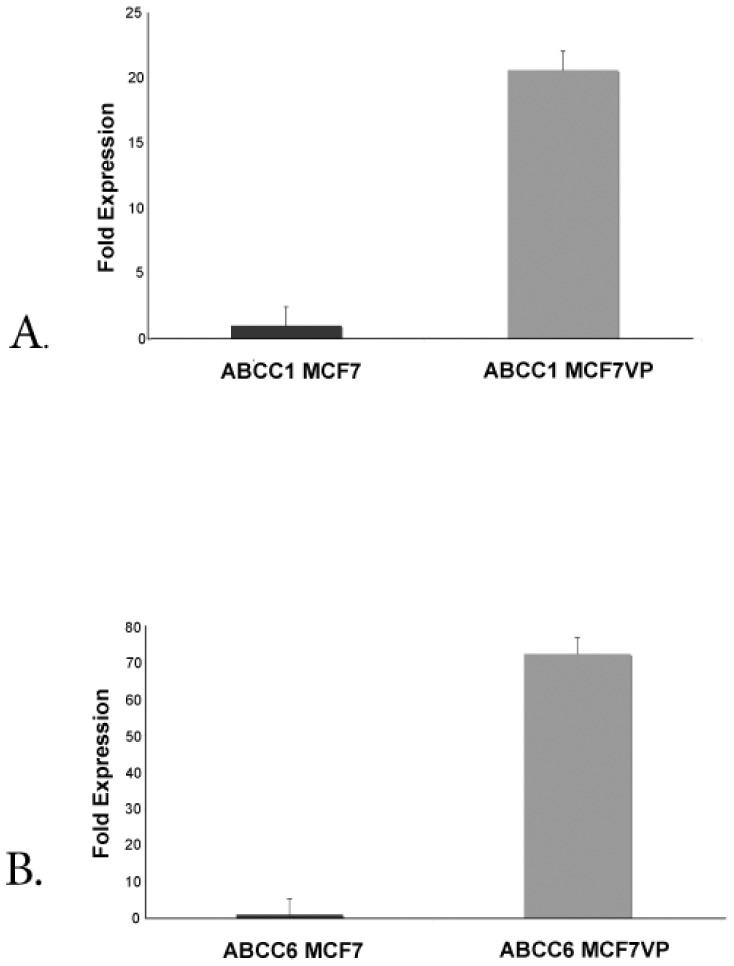
Expression of ABCC1 and ABCC6 in MCF7VP cells, Fig. 3A. Up-regulation of ABCC1 in MCF7VP cells validated by qRT-PCR. The gene expression level of ABCC1 was found to be up-regulated in the MCF7VP cell line using microfluidic arrays, this was validated using quantitative RT PCR with taqman probes against ABCC1. The fold change was calculated using the delta delta CT method. Fig. 3B. Up-regulation of ABCC6 in MCF7VP cells validated by qRT-PCR. ABCC6 up-regulation was validated with quantitative qRT PCR and taqman probes against ABCC6. The fold change in gene expression between the MCF7 and MCF7VP was calculated using the dd CT method.

The ABC (ATP binding cassette) transporters are a diverse family that contain a number of energy dependent efflux pumps. Using TaqMan low density microfluidic arrays which contain the probes and primers (in triplicate) to detect the expression of all 48 human ABC transporters (and also endogenous controls such as beta actin) we found that *ABCC1* and *ABCC6* were highly expressed in the etoposide-resistant cell line compared to the sensitive cell line (Fig. 2). We validated the data using quantitative real time PCR (qRT-PCR) and found that *ABCC1* was 20.5 fold over-expressed (Fig. 3A) and *ABCC6* was 72.5 fold over-expressed in the MCF7VP cell line compared to the parental MCF7 cell line (Fig. 3B). We found that expression of other transporters was not appreciably increased (Fig. 2). We also validated this data using western blotting to show that both ABCC1 and ABCC6 proteins are expressed in MCF7VP cells (Fig. S1).

### MCF7VP Cells have a Distinct miRNA Profile when Compared to MCF7 Drug Sensitive Cells

Using miRNA microfluidic arrays from ABI, we determined the microRNA profile for MCF7VP cells compared to drug sensitive cells (Table 1, Data File S1). Multiple miRNAs showed differential expression among the cell lines including hsa-miR-382, hsa-miR-23b and hsa-miR-885-5p, which were up-regulated (>2-fold increase) in MCF7VP cells and hsa-mir-218, hsa-miR-758 and hsa-miR-548d-5p, which were down-regulated (>2-fold decrease) in MCF7VP cells (Table 1 and Data File S1), suggesting that etoposide resistant MCF7 cells have a miRNA profile that is distinct from the MCF7 drug sensitive cell line. Of particular interest was the 7.79-fold decreased expression of Hsa-miR-218 in MCF7VP cells.

**Table 1:** Micro-RNA expression in etoposide resistant MCF7VP cell line.

Micro-RNA	Log10RQ	neg DDCt
hsa-miR-382	3.2304599	10.7313555
hsa-miR-23b	1.726751554	5.7361445
hsa-miR-885-5p	1.590805204	5.2845405
hsa-miR-184	1.492550819	4.9581465
hsa-miR-342-5p	1.455023517	4.8334835
hsa-miR-484	1.368210079	4.5450955
hsa-miR-491-5p	1.359980822	4.5177585
hsa-miR-330-5p	1.303278812	4.3293985
hsa-miR-29b	1.259166478	4.1828605
hsa-miR-503	1.246293231	4.1400965
hsa-miR-202	1.246093649	4.1394335
hsa-miR-455-3p	1.125521502	3.7389015
hsa-miR-518d-3p	−0.607968759	−2.0196285
hsa-miR-487b	−0.712653445	−2.3673835
hsa-miR-127-3p	−0.810643224	−2.6928985
hsa-miR-523	−0.826782947	−2.7465135
hsa-miR-135a	−0.827046348	−2.7473885
hsa-miR-129-3p	−0.845870657	−2.8099215
hsa-miR-518f	−0.978512601	−3.2505485
hsa-miR-542-3p	−0.991584828	−3.2939735
hsa-miR-198	−1.093894689	−3.6338395
hsa-miR-150	−1.101782879	−3.6600435
hsa-miR-298	−1.380653455	−4.5864315
hsa-miR-31	−1.437412058	−4.7749795
hsa-miR-548d-5p	−1.595658108	−5.3006615
hsa-miR-758	−1.604303389	−5.3293805
hsa-miR-218	−2.346829094	−7.7959975

Only microRNA with log_10_ RQ values greater than 1.12 and less than −0.60 are represented in the table for space considerations. The entire data set is included in the supplementary data (Data File S1) The microRNA expression values are noted as MCF7VP compared to MCF7, Log10RQ values are equivalent to log of fold expression.

### Gene Expression Profiling and Enrichment Analysis Reveals Differences in Gene Expression between the Drug Resistant and Drug Sensitive MCF7 Cells

**Figure 4:**
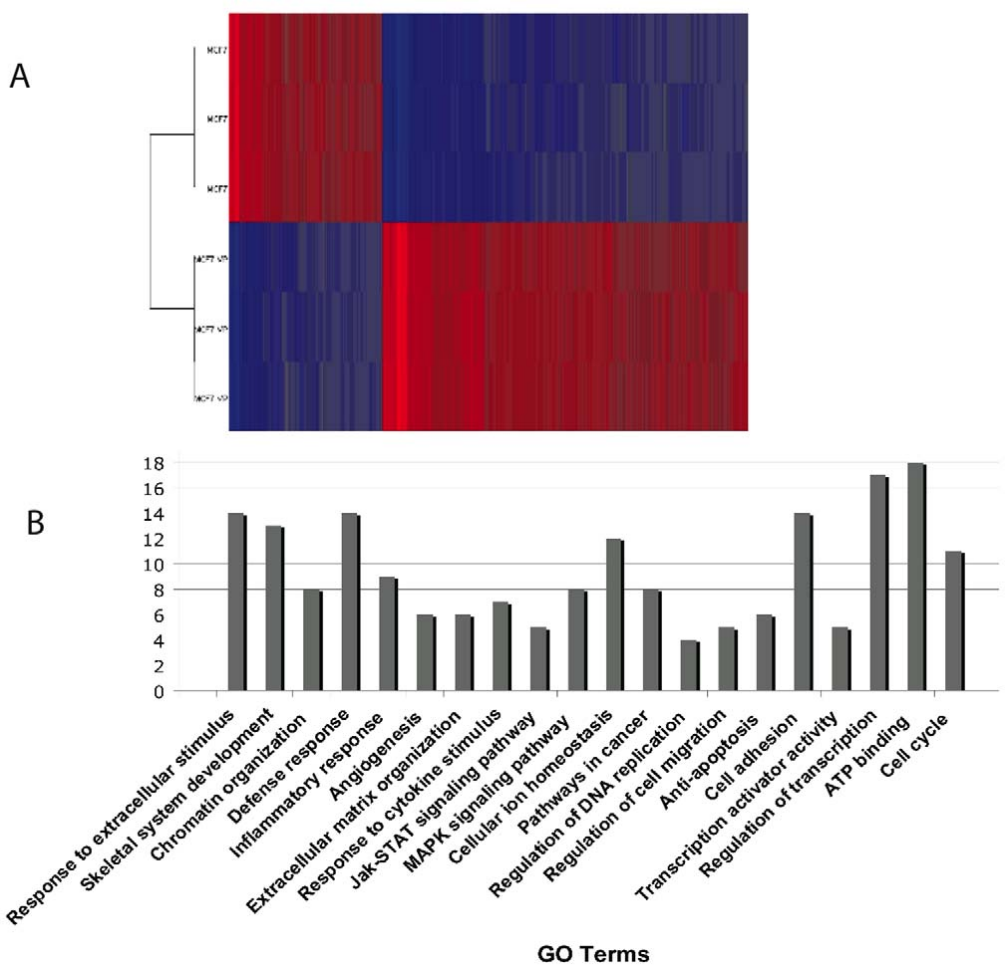
Gene expression microarray data and GO analysis revealed differential gene expression between MCF7 & MCF7VP cell lines. Figure 4A. Heatmap depicting differential gene expression in etoposide resistant and etoposide sensitive MCF7 cells using Affymetrix U133 arrays (>2fold change in expression, p<0.05). Up-regulated genes depicted in red, down-regulated genes in blue (see color bar). Fig. 4B. DAVID analysis of microarray data depicting the number of genes expressed in different cellular mechanisms and pathways (Top 219 genes, p<0.05).

In order to obtain a complete picture of the differences in expression levels between the drug resistant and drug sensitive cell lines, gene expression profiling was carried out using Affymetrix HG-U133 Plus 2.0 arrays and the arrays were run in triplicate which is the industry standard for microarray data. The data was RMA normalized and analyzed with Partek Genomics Suite. The top 5000 differentially expressed genes (≥2 fold change, P<0.05) are displayed as a heat map (Fig. 4A). Twenty-nine genes have greater than 10-fold higher expression in the MCF7VP cell line (Table 2) including *ABCC1* and *ABCC6*, confirming our earlier TaqMan assay results. In addition, the data revealed that topoisomerase 2 (*TOP2A*), which is a target of etoposide, was down-regulated in the MCF7VP cells (The entire data set is deposited in GEO: GEO accession no. GSE28415).

**Table 2:** Correlated copy number and gene expression data.

	Copy Number		Gene Expression	
Gene	Description	p-value	Fold change	p-value
ABCC6	Amplification	0	46.1391	4.31E-06
DKFZp686O24166	Amplification	0	25.2634	1.28E-05
ABCC1	Amplification	9.53E-44	12.89	6.48E-09
NDE1	Amplification	0	11.97	2.72E-09
C16orf63	Amplification	0	9.24178	4.94E-08
IFI6	Amplification	1.31E-09	8.0305	0.000896424
MAFF	Amplification	1.02E-11	6.65262	3.28E-05
FHL2	Amplification	0	6.57373	4.75E-07
PLA2G16	Amplification	1.43E-11	6.47037	1.27E-06
FAM134B	Amplification	7.00E-34	6.45717	1.93E-06
ANKRD57	Amplification	0	6.36357	1.14E-05
UBE2L6	Amplification	0.000104829	6.15093	2.98E-05
GHR	Amplification	0	5.06414	3.09E-06
ODZ3	Deletion	0	−9.0704	3.88E-05
OAT	Deletion	1.15E-26	−6.32636	1.42E-06
GTF2I	Deletion	1.50E-07	−6.05823	1.46E-05
ZNF829	Deletion	0	−5.49106	0.000354608
SEPT11	Deletion	5.48E-06	−5.19448	0.000214344

### Functional Analysis of the Microarray Data Predicted Several Possible Pathways of Etoposide Resistance

**Figure 5:**
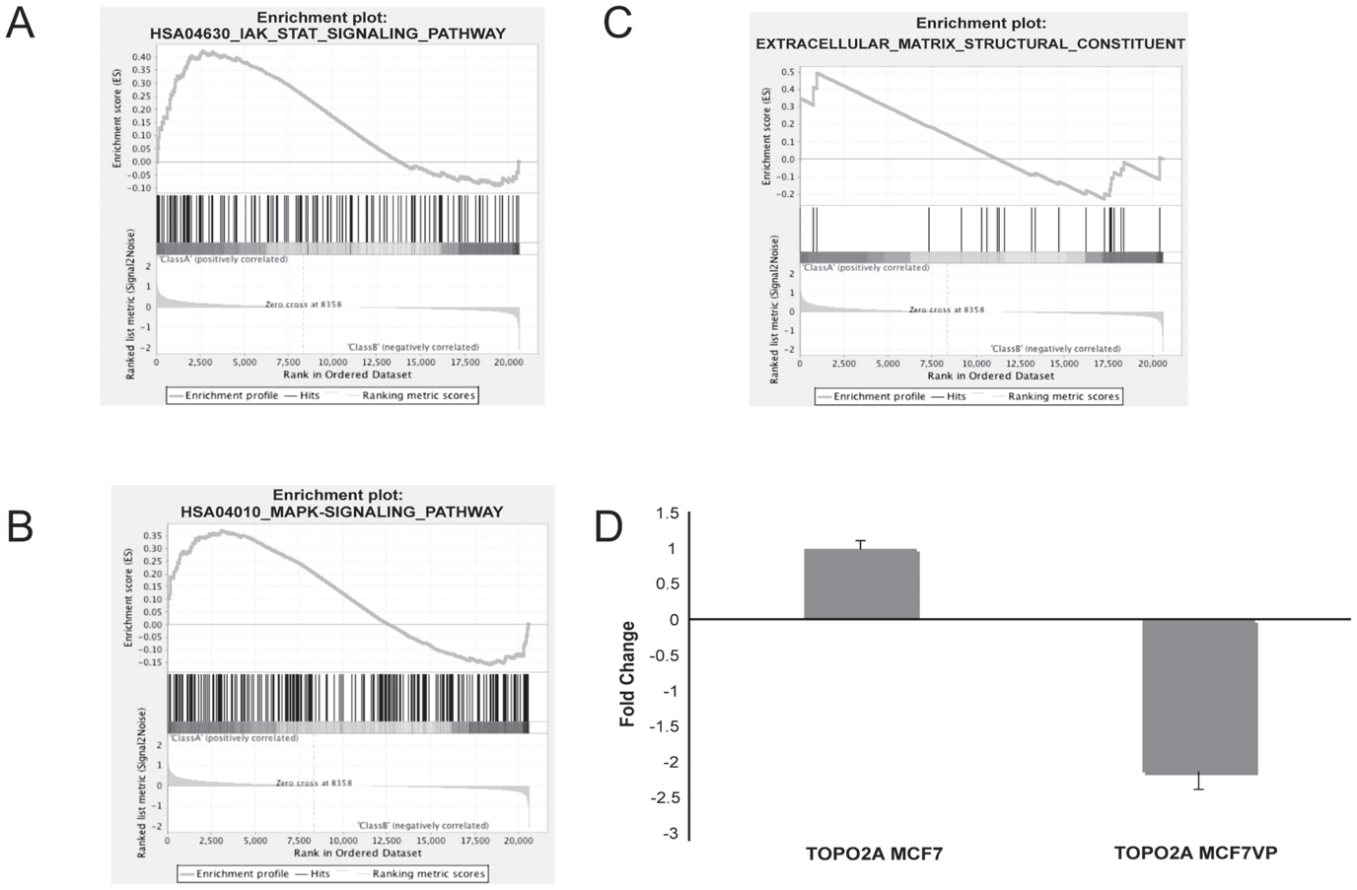
GSEA analysis of microarray data showing enrichment of the JAK-STAT and MAP Kinase pathways and upregulation of ECM structural component genes along with qRT-PCR validation of the down-regulation of topoisomerase 2 gene expression in MCF7VP cells. Fig. 5A. Gene set enrichment analysis of microrray data depicting the enrichment of genes in the JAK-STAT signaling pathway. The GSEA software was used to calculate the enrichment levels. Fig. 5B. Gene set enrichment analysis of microrray data depicting the enrichment of genes in the MAP kinase signaling pathway. The GSEA software was used to calculate the enrichment levels. Figure 5C. Gene set enrichment analysis depicting enrichment of ECM (extra-cellular matrix) genes in MCF7VP cells. The GSEA algorithm was used to calculate the enrichment levels. Figure 5D. Down-regulation of TOPO2A (the drug target of etoposide) in MCF7VP cells. The microarray data was validated by qRTPCR as depicted in the bar chart which shows differences in fold change.

Gene Ontology (GO) analysis of the microarray data revealed a number of gene clusters that could be associated with drug resistance. DAVID, an annotation and integration tool, was used to explore the differentially expressed genes (top 219 genes, p<0.05) in etoposide-sensitive and resistant cell lines. We identified possible indicators in pathways that might be associated with drug resistance including extra-cellular matrix (ECM) organization, JAK-STAT signaling pathway, MAP kinase signaling pathway, and ATP binding (Fig. 4B). GSEA (Gene Set Enrichment Analysis) validated that genes involved in the JAK-STAT pathway (Fig. 5A), MAP Kinase pathway (Fig. 5B), and ECM constituents (Fig. 5C) were enriched in the drug resistant cells. We also found that extra-cellular matrix (ECM) genes are upregulated in etoposide resistant cells. The extra-cellular matrix primarily provides support to animal cells and tissues. Analysis showed that over 66% of extra-cellular region genes are up-regulated in the MCF7VP cells (Fig. S2). These genes include: collagens (particularly collagen type 3A1 and collagen type 12A1), fibronectin and matrix-gla protein, which are involved in remodeling of the ECM. Some studies have shown that the ECM may mediate drug resistance by either disrupting integrin signaling or by the formation of an actual physical barrier by remodeling of the ECM [12] however, very little is known about this mode of drug resistance. IPA analysis validated that integrin signaling was one of the pathways upregulated in the microarray dataset (Fig. S3). In addition, we validated the fact that *TOP2A* gene was down-regulated using qRT-PCR, which showed that the gene expression of *TOP2A* was decreased by 2.1 fold in the MCF7VP cell line (Fig. 5D). This may be indicative of a possible modulation of drug resistance through the downregulation of TOP2A where the MCF7VPcells may circumvent drug sensitivity by down-regulating the drug target – in this case Topoisomerase 2A.

### Copy Number Analysis Identified Differences in Copy No. of Genes between the MCF7 and MCF7VP Cell Lines

**Figure 6:**
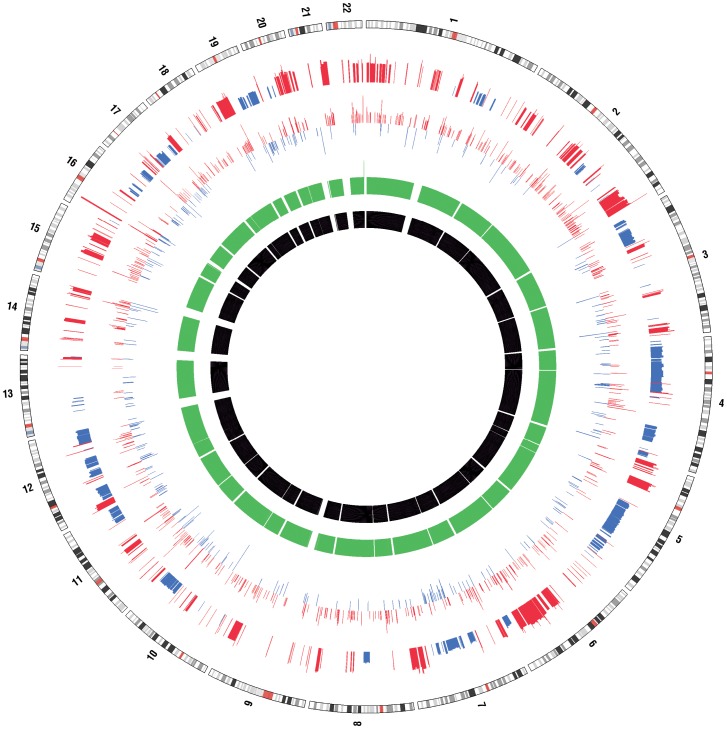
Circos plot incorporating differential gene expression and LOH. Chromosome numbers and bands are identified in the outer-most ring. Other tracks from outer to inner represent: amplifications (red) and deletions (blue) in MCF7VP compare to MCF7 using Affy 6.0 SNP data (Partek); differential expression (p<0.05) between MCF7 and MCF7VP (AffyU133), ln(MCF7/MCFVP) values plotted; LOH in MCF7 derived from Affy 6.0 SNP data (green); LOH in MCF7VP derived from Affy 6.0 SNP data (black).

As expected, the number of copy number changes detected with the Affymetrix 6.0 chip varied greatly between the 2 cell lines. Numerous amplifications and deletions were detected on chromosomes in the MCF7VP cells when compared to the baseline of the parental MCF7 cells (Table 2). The copy number analysis was further verified by analyzing loss of heterozygosity (LOH) of the SNPs on different chromosomes. LOH is a form of allelic imbalance that can result from the complete loss of an allele or from an increase in copy number of one allele relative to the other. We compared the copy number analysis with the gene expression data and found the both *ABCC6* and *ABCC1* were amplified in the chromosomal DNA and also at the RNA level. The *GHR* gene or growth hormone receptor involved in various signal transduction pathways (eg;cellular growth) was also up-regulated at both DNA and RNA levels (Table 2). The *MAFF* oncogene which is thought to be associated with cellular stress was upregulated and showed an increase in copy number. A Circos plot was generated incorporating differential gene expression and loss of heterozygosity (Fig. 6).

## Discussion

In this study we have described gene and miRNA indicative of resistance in the MCF7VP etoposide-resistant breast cancer cells. Gene expression data and copy number analysis (Table 2) revealed that the transporter genes *ABCC1* and *ABCC6* are significantly up-regulated in MCF7VP cells. It has been previously found that the transfection of MRP1/ABCC1 expression vectors into HeLa cells resulted in these cells becoming moderately cross-resistant to several drugs including etoposide [13] and that *ABCC6* transfected Chinese hamster ovary (CHO) cells show a low level of resistance to etoposide [14]. Our findings are consistent with these studies. However, we did not observe significant up-regulation of other ABC transporters including *ABCB1* or *ABCC3* [15] that are thought to be associated with etoposide resistance. It was recently shown that *ABCC6* was five-fold up-regulated in gemcitabine resistant A549 non-small cell lung cancer cells [16] suggesting that it also may be a factor in influencing gemcitabine resistance.

It has been previously reported that Hsa-miR-326 can target *ABCC1* 3′UTR [17] in MCF7VP cells and accordingly we found that this miRNA was down-regulated in the etoposide resistant cells when compared to the sensitive cells (Data File S1). Other transporters such as ABCB1 (P-gp) are known to be regulated by microRNA. It was previously found that antagomirs of hsa-miR-451 and 27a can target *MDR1* in A2780DX5 cells [18]. Taken together, this suggests that microRNA’s and their antagomirs could be used to modulate the expression of ABC transporters.

We also found a set of extra-cellular matrix genes were up-regulated in the etoposide resistant cells. It has been proposed that the extra-cellular matrix may be an important factor in contributing to drug resistance either by the disruption of integrin signaling or by the formation of an actual physical barrier achieved by remodeling of the ECM, [12] however, very little is known about this particular mode of drug resistance. Currently there is scant evidence to support the role of ECM in drug resistance, but our findings suggest that this may be a valid factor in etoposide resistance.

Finally, we found that the copy numbers of both *ABCC1* and *ABCC6* were increased significantly in the MCF7VP cell line and this data correlated well with our gene expression data (Table 2). As expected we found differences in copy number in a several genes between the drug resistant and drug sensitive cell lines which correlated for the most part with the gene expression data (Fig. 6). This would suggest that, along with upregulation of gene expression, alteration in gene copy number may be a mechanism of activating or down-regulating genes in chemoresistance. This was also suggested in a previous study by Yasui et al 2004 [19].

In the current study we identify a number of molecular indictors of etoposide resistance in the MC7VP etoposide resistant cell line. Our work may serve as a model on which to base future investigations of etoposide resistance in diverse cancer cell lines and also in clinical patient samples.

## Materials and Methods

### Cell Lines and Culture Conditions

The MCF7VP (etoposide resistant) cell line was obtained from Rob Robey -originally sourced from Dr. Kenneth Cowan, University of Nebraska Medical Center, NE, USA [20] and was cultured for at least 8 passages in 4 µM etoposide in IMEM with 10% FBS and 1% Penicillin-Streptomycin. The paired MCF7 parental cell line was also cultured in IMEM with with 10% FBS and 1% Pen-Strep. MCF7 was obtained from the DTP (Developmental Therapeutics Program) NCI-Frederick that was also originally sourced from Dr. Cowan [20].

### Cellular Cytotoxicity Assay to Determine Sensitivity to Etoposide

The cellular cytotoxicity kit from Dojindo (Rockville, MD) was used for this assay as per manufacturers instructions.

The cellular cytotoxicity assay allows sensitive colorimetric assays for the determination of cell viability. A highly water-soluble tetrazolium salt, WST-8*, is reduced by dehydrogenase activities in cells to give a yellow-color formazan dye, that is soluble in the tissue culture media. The amount of the formazan dye, generated by the activities of dehydrogenases in cells, is directly proportional to the number of living cells hence the toxicity of etoposide can be determined by measuring absorbance of the formazan dye. Briefly 10,000 cells per well of a 96 well plate were plated and preincubated at 37 degrees C for 24 hrs. after which 100 µl of various concentrations (0–100 µM) of etoposide was added. After 72 hrs incubation 10 µl of CCK8 reagent was added. Two hours later the absorbance was measured at 450 nm. Each experiment was carried out twice and the data pooled. Calculations were carried out with MSExcel and GraphPad prism and R.

### Isolation of Total RNA, Including the miRNA Fraction

RNA was isolated from MCF7 and MCF7VP cells using the mirVana kit from ABI as per manufacturers instructions (Applied Biosystems (ABI), Carlsbad, CA) and analyzed using an Agilent bioanalyzer to determine the quality and quantity of the RNA.

### Reverse Transcription and Running ABC Transporter TLDA (Taqman Low Density Arrays)

The RNA was reverse transcribed into cDNA using the high capacity reverse transcriptase kit (ABI). The cDNA was used to run TLDA- ABC (ATP binding cassette) transporter arrays or individual reactions with the relevant primers on a 7900HT real time PCR machine. The data was analyzed using Microsoft excel and R-Bioconductor to generate heat maps of the ABC array data.

### Conversion of RNA to cDNA and Running miRNA Array

The isolated RNA was converted to cDNA using human miRNA primer pools A & B respectively (ABI) and the cDNA was used to run the real-time reaction in two human miRNA TLDA (Taqman low density array ABI cards version 2.0) on a 7900HT real time PCR machine. The data was imported into the StatMiner software (Integromics) and analyzed using the default settings. We used the appropriate protocols to validate some of the reactions in single-tube reaction mixes to verify expression of miRNA (Applied Biosystems protocol. Part No: 4364031, Rev C 12/2009).

### Reverse Transcription and qRTPCR

The RNA was reverse transcribed into cDNA using the high capacity reverse transcriptase kit from ABI. Briefly, we made a reaction mixture of the following components- 10× rev transcription buffer (10 µl) 25× dNTP’s, (4 µl), 10× random primers (10 µl), multiscribe reverse transcriptase (5 µl), Nuclease Free Water (21 µl) and 30 ng of RNA. The PCR reaction was carried out on a thermal cycler with the following parameters: 25°C for 10 min, 37°C for 120 min, 85°C for 5 min to synthesize the cDNA. The real time reaction was carried out using taqman primers and probes specific for a particular gene. We added 2X Taqman Universal PCR master mix (10 µl) RNAase free water (8 µl), 20X target primers and probes (1 µl), cDNA sample 30 ng in a microfuge tube. This was centrifuged for 1 min at 1000 rpm. The PCR cycle was carried out in a 7900 real time instrument (ABI) under the following conditions: 50°C (2 mins) hold, 95°C (10 mins) hold. Then 40 cycles, 95°C for 15 secs and 60°C for 1 minute. The qRTPCR data was analyzed using RQ manager (ABI).

### Affymetrix U133 plus 2 Arrays

Affymetrix HG-U133 Plus 2.0 arrays were run in triplicates with RNA isolated from MCF7 and MCF7VP cells at the Laboratory of Molecular Technology, Frederick, MD. Briefly, for each technical replicate (3 replicates), 100 ng of total RNA were amplified and labeled using the message amp II-biotin enhanced reagents (Ambion) according to the protocol provided by the supplier. Arrays were hybridized with 11 µg of labeled cRNA, washed, stained, and scanned according to the protocol described in Affymetrix GeneChip Expression Analysis Manual (Fluidics protocol FS450_0001). Arrays were scanned using an Affymetrix GeneChip scanner 7G, the .CEL files for each array was imported into Partek Genomics Suite and normalized using the RMA (Robust Multichip Averaging) algorithm ANOVA was used to determine significantly differentiated probe sets between samples Data was deposited in GEO -accession no. GSE28415, http://www.ncbi.nlm.nih.gov/geo/query/acc.cgi?acc=GSE28415. Gene ontology (GO) analysis was carried out using DAVID v6.7 [21], [22], gene set enrichment analysis was done with GSEA [23], [24] and pathway maps were constructed with IPA (Ingenuity Pathway Analysis) software. All microarray experiments were done in compliance with MIAME guidelines.

### Determination of DNA Copy Numbers Using the SNP 6.0 Array

We followed the manufacturer’s instructions for the Affymetrix Genome-wide Human SNP array 6.0. A SNP array can be used to generate a virtual karyotype using software to determine the copy number of each SNP on the array and then align the SNPs in chromosomal order. To detect copy number variations genomic DNA from the MCF7 and MCF7VP samples was labeled, fragmented and hybridized to Affymetrix SNP6.0 arrays according to Affymetrix protocols.

### Data Analysis

#### Analysis of miRNA data

The miRNA data obtained was analyzed using the StatMiner software suite (from Integromics) to determine differential expression of miRNA. Briefly, the data was imported into StatMiner, visually inspected for any anomalies, filtered and unexpressed detectors were flagged and the data was normalized with the help of the appropriate endogenous control. We then performed hierarchical clustering to generate heatmaps and also bar charts of the resulting analyzed data to determine miRNA expression profiles both in StatMiner and in R-bioconductor.

#### Analysis of microarray data

Using the gene expression workflow in Partek (Partek® software, version 6.4 Copyright © 2010,Partek Inc., St. Louis, MO, USA,) we converted the .CEL files generated by the Affymetrix data file into.FMT files (which can be later converted to .XLS files) and normalized the files using RMA normalization procedure. We identified differentially expressed genes using ANOVA (analysis of variance) analysis, filter (<2 fold change, p value >0.05) and displayed the data as a heat map and exported to an excel file to create a gene list of differentially expressed genes between the drug sensitive and drug resistant cells.

#### Quantitative Real Time PCR data analysis

Relative quantification of the expression level of each transcript in each sample was calculated using the delta delta CT method in RQ manager (ABI-Prism). Human beta actin was used as the endogenous reference gene.

#### DAVID (v6.7 from NIAID, NIH)

We imported the filtered microarray gene list using affymetrix gene identifiers into the DAVID workflow and conducted a functional annotation clustering analysis. This yielded functional annotation clustering scores for groups of genes to discover enriched functionally related gene groups. This was then compared between the drug sensitive and drug resistant cell lines. Using DAVID gene ontology analysis we also investigated the function of these genes and redirected to relevant literature.

#### GSEA (Broad Institute)

We used GSEA analysis to also calculate gene enrichment scores and also graphically represented these scores. Briefly, we imported the filtered microarray data and ran the analysis using a fixed number of permutations.

#### Copy number data analysis

Paired copy number analysis was performed on DNA from both cell lines with the Partek Genomics Suite, using SNP 6.0 intensity data (Affymetrix ‘.cel’ files) with the DNA of MCF7 used as a reference. Differences were analyzed between the paired samples based on the gene involved and the base pair start and end of the region of change. Changes were described as either amplification (if the average intensity value was over the diploid value of 2) or deletion (if under 2). The genomic segmentation algorithm was used with default parameters to identify regions of amplification or deletion and a t-test was used to assess significance of the regions. The copy number and gene expression data were displayed as a circos plot [25].

## Supporting Information

Figure S1 **Western blot depicting ABCC1and ABCC6 protein expression in MCF7 and MCF7VP cells.** The blot shows expression of ABCC1 and ABCC6 proteins in MCF7VP cells compared to parental MCF7 cells. Antibodies used were, ABCC1- Primary antibody: MRPm5 (Abcam), secondary antibody: Anti-mouse IgG whole (Sigma). ABCC6- Primary antibody: MRP6 (Santa Cruz), secondary antibody: Goat anti-rabbit IgG-HRP (Santa Cruz). Beta actin- Primary antibody: Beta-actin A4700 (Sigma), secondary antibody: IgG (mouse), Cell Signaling. Western blotting was carried out by standard methods.
(EPS)

Figure S2 **Forest plot of MCF7VP vs MCF7 cells**. The figure depicts over 66% upregulation of extracellular region genes (among others). The plot was constructed using Partek genomics suite.
(EPS)

Figure S3 **Ingenuity Pathway Analysis (IPA).** The figure shows a bar graph demonstrating up-regulation of genes in numerous pathways in the MCF7VP cell line including integrin signaling.
(EPS)

Data File S1 **MicroRNA Array Data.** The .xls data file contains the results of the differential expression of miRNA between the MCF7VP (MCF7.VP) and the parental MCF7 cell line (MCF7.EB).
(XLS)
